# Development of chimeric antigen receptor (CAR) T-cell immunotherapy for glioblastoma targeting epidermal growth factor receptor variant III (EGFRvIII)

**DOI:** 10.1186/2051-1426-3-S2-P119

**Published:** 2015-11-04

**Authors:** Anthony K Park, Saul Priceman, Ethan Gerdts, Wen-Chung Chang, Sarah Wright, Stephen J Forman, Christine E Brown

**Affiliations:** 1 City of Hope National Medical Center, Duarte, CA, USA; 2 Beckman Research Institute, City of Hope National Medical Center, Duarte, CA, USA

## 

Glioblastoma multiforme (GBM) is the most common brain tumor, with a poor prognosis of less than 14 months after initial diagnosis. Gene amplification and mutation of epidermal growth factor receptor (EGFR) are frequently observed in primary GBM. The most common variant of EGFR, known as EGFRvIII, is expressed in approximately 30% of GBM patients, but is absent on normal cells, making it a desirable target for cancer immunotherapy. In addition to the surge of adoptive cell immunotherapies for the treatment of advanced cancers, chimeric antigen receptors (CARs) have come to the forefront as a promising therapeutic strategy. We have engineered genetically modified T cells to express CARs that specifically target tumor cells expressing EGFRvIII and/or amplified EGFR for the treatment of malignant gliomas.

Our studies have focused on optimizing the EGFRvIII-CAR structural design, including the antigen-targeting domain (scFv), extracellular non-signaling linker, and intracellular co-stimulatory signaling domains, to improve overall CAR T cell specificity and efficacy. Thus, we have compared five different scFvs for targeting EGFRvIII, and four different linkers with varying lengths. CAR activity was evaluated using a variety of *in vitro* functional assays, including CD107a degranulation, IFN-γ production, PD-1 induction, T cell proliferation and tumor killing assays. Using these assays, we have defined CARs capable of mediating highly-specific activation and killing of target tumor cells expressing EGFRvIII and/or amplified EGFR, with minimal targeting of endogenous EGFR-expressing cells. We further validated our EGFRvIII-specific CAR T cells using preclinical human xenograft models of GBM. Our *in vivo* tumor studies show dose-dependent killing by locally administered EGFRvIII-CAR T cells against subcutaneous tumors expressing EGFRvIII. Combined, these studies demonstrate potent anti-tumor activity of EGFRvIII-specific CAR T cells.

